# RegistrAME: the Spanish self-reported patient registry of spinal muscular atrophy

**DOI:** 10.1186/s13023-024-03071-7

**Published:** 2024-02-19

**Authors:** Maria Grazia Cattinari, Mencía de Lemus, Eduardo Tizzano

**Affiliations:** 1 Fundación Atrofia Muscular Espinal España (FundAME), Madrid, Spain; 2 SMA Europe, Freiburg, Germany; 3 Committee of Advanced Therapies at the European Medicines Agency, Amsterdam, The Netherlands; 4 Department of Clinical and Molecular Genetics and Rare Diseases Unit and Medicine Genetics Group, VHIR, Hospital Valle Hebron, Barcelona, Spain

**Keywords:** RegistrAME, Spinal muscular atrophy, Patient registry, Real-life, Self-reported registry

## Abstract

**Background:**

Spinal Muscular Atrophy (SMA) is a rare neuromuscular disorder characterized by progressive degeneration of motor neurons and muscle weakness resulting in premature death or severe motor disability. Over the last decade, SMA has dramatically changed thanks to new advances in care and the emergence of disease-specific treatments. RegistrAME is a self-reported specific disease registry with an accurate curation system. It has collected data on SMA patients in Spain since 2015, gathering demographic, clinical, and patient-reported outcome data, all of which are patient-relevant. RegistrAME is part of the TREAT NMD network. This study aims to describe the advantages and disadvantages of a self–reported SMA registry, as well as the different variables of interest in the health status of RegistrAME patients.

**Results:**

In total, 295 living patients with a confirmed diagnosis of SMA-5q were included (aged 1 to 77 years; mean 20.28). Half of the patients (50.2%) were ≥ 16 years old; 22.03% were type 1, 48.47% were type 2, 28.82% were type 3, and 0.7% were type 4. All functional statuses (non-sitter, sitter, and walkers) could be observed in each SMA type. Adult patients harbored the least aggressive SMA types, however, they presented the greatest level of disability. Patients with SMA type 1 had scoliosis surgery about five years earlier than patients with SMA type 2. None of the type 1 patients who achieved ambulation were wheelchair-free outdoors. This was also evident in 62.5% of type 2 walker patients and 44% of type 3 walker patients. Of the SMA type 1 patients, 40% had a gastrostomy (of which 84% had two *SMN2* copies). One in five children with SMA type 1 (one to seven years of age) were ventilation-free.

**Conclusions:**

The information provided by RegistrAME in a “real-world” setting allows better management of family expectations, an adequate approach to the disease and patients’ needs, as well as a better understanding of the impact of the disease. It also helps monitor the evolution of care, which will result in the need for updated guidelines.

## Background

Spinal Muscular Atrophy (SMA) is a severe progressive neuromuscular disorder that results in premature death or severe motor disability [1]. SMA is characterized by degeneration and death of alfa motor neurons mainly in the anterior horn of the spinal cord and progressive muscle weakness, muscle atrophy, and paralysis [2].

The 5q SMA or SMN-dependent SMA (hereinafter SMA) is caused by a shortage of survival motor neuron (SMN) protein, due to bi-allelic pathogenic variants of the *SMN1* gene on the long arm of chromosome 5 [3, 4]. It has an incidence of 1 in 6,000 to 10,000 live births [2, 3].

The absence of the *SMN1* gene is partially compensated by a paralogous gene, *SMN2*, which produces a functional protein at levels insufficient to fully compensate for the absence of the *SMN1* gene.

Usually, a higher number of copies of the *SMN2* gene ameliorates the phenotype [5] and *SMN2* is, so far, the only validated phenotype modifier of the disease.

SMA presents a continuous spectrum of severity; nevertheless, five phenotypes (from the most severe congenital form—type 0—to the mildest form—type 4) are traditionally recognized, based on both age of onset and the greatest milestone achieved by patients [1, 6]. SMA phenotypes vary in severity, however, in all cases, the impact of muscle weakness is always devastating across the spectrum [7].

In December 2016, the Food and Drug Administration (FDA) approved nusinersen, the first treatment for SMA [8]. In May 2019, the FDA approved onasemnogene abeparvovec-xioi, the first genetic therapy for SMA [9]. Risdiplam, the first oral treatment, was approved by the FDA in August 2020 [10]. The European Medicines Agency (EMA) also approved these treatments in May 2017, May 2020, and March 2021, respectively [11–13].

Over the past decade, there has been an increase in patient survival for all types of SMA due to a more proactive approach and new advances in care, such as the introduction of non-invasive ventilation and enteral feeding [14]. Progression in care and the effective management of complications, in addition to the availability of disease-modifying therapies (DMT), translate into an even more significantly increased survival [15]. This changes the phenotypes that modify the trajectories of the disease, which are currently evolving but may also generate new complications [16].

### Why an SMA Registry?

SMA is classified as a rare disease; there are between 5,000 and 8,000 different rare diseases, affecting between 6% and 8% of the world’s population [17]. Long-term prospective registries are useful instruments for creating a comprehensive and broad knowledge base for these diseases [18]. They collect data for epidemiological and clinical research, and subsequent real-life observational studies [18]. Additionally, there is a need to monitor the long-term safety and efficacy of new DMTs in the real world [19]. Patient registries have been considered a global priority in the field of rare diseases [20]. The development of records to ensure the continuity of care in neurological disorders is a core competency identified by the WHO Global Health Report [19].

This study aims to perform a descriptive and exploratory analysis of the different variables of interest in the health status of patients pertaining to the Spanish Registry of Spinal Muscular Atrophy (RegistrAME). Furthermore, we aim to identify those aspects or variables that are most relevant or that have a greater impact on the patient’s symptoms, evolution, and quality of life.

## Materials

### RegistrAME

FundAME’s Registry (RegistrAME: *Registro Nacional de Pacientes Atrofia Muscular Espinal)* is a voluntary patient-reported database launched by FundAME, the Spanish SMA Patient Organization. The registry was launched in 2015 with a view to increasing the knowledge of the pathology aligned with the Treat NMD Guidelines. In 2017, FundAME introduced modifications to the dataset to guarantee its independence, ensure patient relevance, and its compatibility with the Treat NMD dataset. In May 2020, a new module for patient-relevant outcomes (PROS), PROfuture, was incorporated into the registry. RegistrAME is currently part of the CORE group of TREAT-NMD registries. The dataset follows the international guidelines of Treat-NMD, however, its formulation involved patients, and the set of items was assessed for comprehensibility by the same patients.

RegistrAME is a specific independent record of genetically confirmed SMA patients. This disease-specific SMA registry is a prospective long-term selfreported patient registry, supervised by expert SMA clinicians.

The objective of RegistrAME is to collect longitudinal data from all types of SMA patients in Spain. Being an online registry, it allows the inclusion of patients from all over the Spanish territory, including patients with or without specialized clinical follow-up, as well as patients with or without treatment. All these patients provide epidemiological and clinical data in the “real world”.

### Information technology platform

The registry Information Technology (IT) platform is hosted on Azure, a set of cloud services owned by Microsoft, which has its own source code. The data is stored in Microsoft Azure data centers located in Europe, ensuring compliance with the EU General Data Protection Regulation.

The platform is made up of two servers, one in which the database containing personal information is housed, and a second one in which the patient’s health database is housed. Both servers are protected by passwords and other security measures and also by Azure’s security infrastructure. It also has storage containers in Azure where the patient’s genetic reports, audit records, and certificates are kept.

The registry, in addition to a username and password, incorporates a verification system or two-factor authentication for accessing the database. Patients’ personal data are collected in tables protected with Row-Level Security, which prevents data from being requested directly from tables or by unidentified users in personal databases, and Dynamic Data Masking which masks personal data, both for the account and for the patients created in the account.

### Relevant information about SMA

The information requested by RegistrAME includes health data compatible with the items established by TREAT-NMD (2.0 version), as well as items related to daily life, function and independence, obtained with the collaboration of both patients and clinical experts.

The clinical dataset contains relevant clinical information such as genetic data (including *SMN1* pathogenic variants and *SMN2* copy number), age of onset and diagnosis, SMA type, best milestones achieved and eventual age of loss, current motor function, surgical intervention for scoliosis, swallowing and feeding issues, respiratory care, current medication, participation in clinical trials, comorbidities… among others. It also includes relevant information for the patient, such as the impact of scoliosis surgery on function, the impact of fatigue and fatigability, time spent in care, pain, etc.

### Source of information

Once diagnosed with SMA, patients are informed by their specialist doctors about the existence of the FundAME registry. Additionally, FundAME actively promotes the registry through its website, social media channels, regional delegates, conferences, and collaborations with other patient associations.

All data included in the registry (demographic characteristics and pathological, clinical, and therapeutic data of the disease, as well as the Patient-Relevant Outcomes module -PROfuture-) are entered by the patients or their caregivers. This patient-centered registry, designed with active patient involvement, employs terminology accessible to patients. Furthermore, it offers support from the registry manager for any queries. Patients will be contacted (by mail, phone) to inform them about necessary updates or any data that generate discrepancies.

Patients are encouraged to update their data every six months.

### Patient’s access to registry

The first step of the process is to fill out a form located on the registration website. Access URL: https://www.registroame.org.

To initially enroll in the registry (pre-registration), it is necessary to download an IC (Informed Consent) that patients must sign and send to FundAME by post or courier with a copy of their Identity Card.

Once the data manager has received the IC signed by the patient or the parent/tutor, the patient’s account in the registry is activated. Access to this account will be restricted to the patient or their corresponding caregiver via access codes (User Name and Password) that are created by each user.

**Fig. 1:**
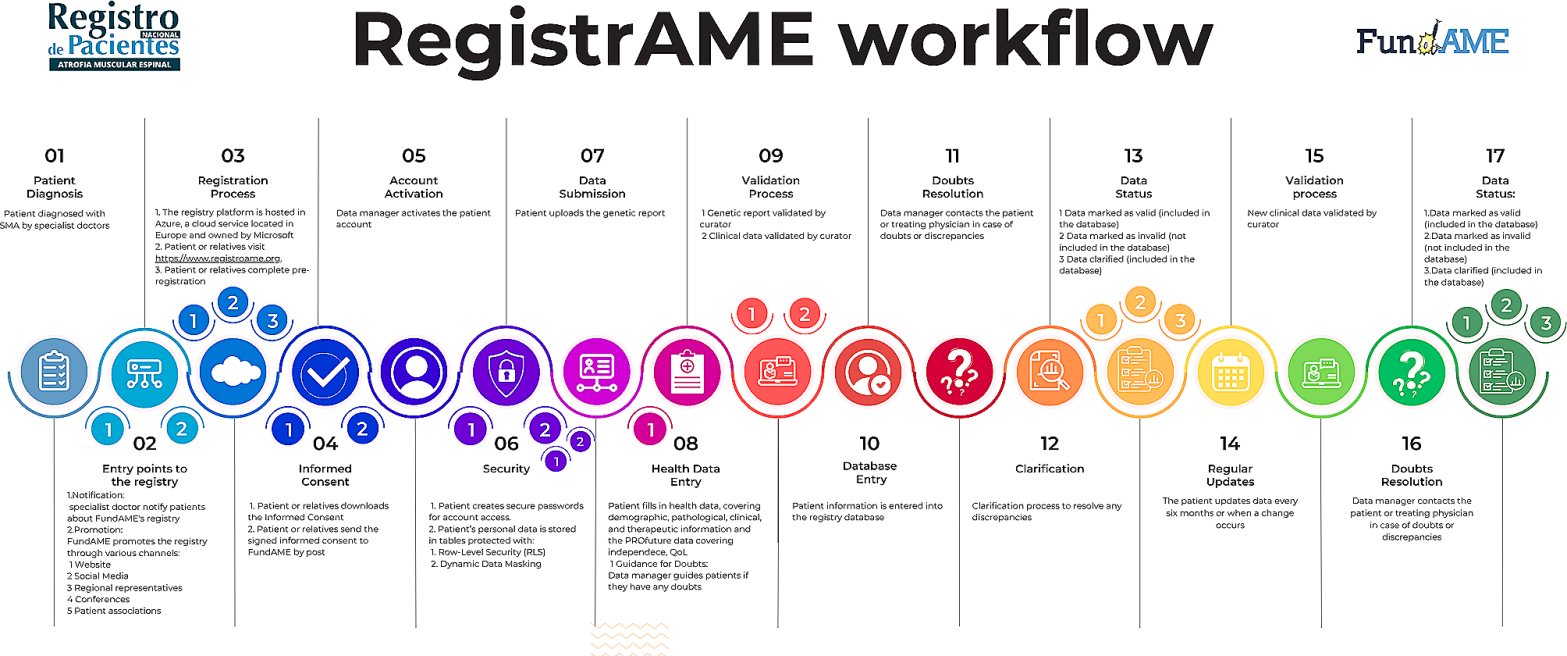
RegistrAME workflow

Once the patient’s account is active, the patient can upload their genetic report, as well as fill in all their health data. Outlines the sequential steps users follow during the registration process in Fig. 1.

### Data curation

Clinical data, such as the type of disease-causing variants, the number of *SMN2* copies, and the Forced Vital Capacity (FVC) value, are confirmed with reports uploaded by patients to the platform. Values from other diagnostic tests, as well as motor scales, are not recorded. All other data is curated in its entirety (excluding Patient-Reported Outcomes - PROs).

Once the registry curators (physicians with experience in SMA) validate the genetic report, as well as the clinical data, the patient’s information is entered into the registry database.

In the case of any ambiguous information that may raise doubts for the curator, or any discrepancy in the information provided, patients or treating physicians are contacted for data clarification.

Health data marked as invalid is not included in the database (for statistical extraction) until the Curator has rectified the inconsistency. The curation process is limited to health data and is not performed in the PROfuture module.

This meticulous process ensures a thorough quality assessment of the evaluated data, with the purpose of guaranteeing data integrity and accuracy. By confirming key clinical parameters directly with patient-uploaded reports and engaging in proactive communication to curate the remaining information, we established a robust framework to maintain the overall quality of the dataset.

### Ethics approval

RegistrAME was approved by the ethics committee (CEIm): Hospital Universitario 12 de Octubre Committee approval No. CEIm: 20/413.

## Methods

All data comes from RegistrAME: the Spanish Registry of Spinal Muscular Atrophy. The dataset analyzed reflects the registry’s dataset up to July 2022.

### Inclusion criteria

1. Patients with a confirmatory genetic report of 5q SMA.
2. Residents in Spain.
3. Their file has been validated by the curators.

### Exclusion criteria

1. Deceased patients.
2. Non-5q SMA patients.

The Spanish consensus on SMA was followed to classify patients. Individuals under the age of 16 were categorized as children while those aged 16 years or older were classified as adults. Consistent with this consensus, patients were further categorized into four types based on the age of symptom onset and motor milestones achieved. Type 1 includes those initiating symptoms before six months of age and failing to achieve sitting. Type 2 comprises individuals manifesting symptoms between 6 and 18 months, achieving independent sitting but not independent walking. Type 3 involves those with symptom onset after 18 months who achieve independent walking. Type 4 encompasses those whose clinical onset occurs from the age of 20 years onwards.

Thirteen deceased patients were not included in this extraction, as some had passed away prior to the implementation of the current data curation process. Therefore, their data did not undergo the same current data curation process. Additionally, for data protection reasons, personal information, including date of birth, was removed from deceased patients, which limits the analysis.

A total of five patients were deregistered due to relocation to another country or at their request. In addition, four patients were not considered for this extraction because they temporarily moved to another country.

### Statistical methods

The data was summarized by using mean (standard deviation) and median (1st and 3rd quartiles) in the case of numerical variables, and by absolute and relative frequencies in the case of categorical variables.

In addition, association graphs were drawn for different contingency tables, as well as a general study of the association between the different variables using the Goodman-Kruskal tau method. To characterize the patients’ evolution until they needed assisted ventilation, a survival analysis was performed using Cox regression. A similar analysis was also performed for surgical data.

For all analyses, the significance level was set at α = 0.05, and 95% confidence or credible intervals were estimated for all parameters of interest.

All analyses were performed with R software (4.2.1 version) and the R packages brms (2.17.0 version), survival (version 3.3-1), and clickR (0.8.1 version).

## Results

### Curation process

All health data underwent curation. The most common discrepancies observed were:

- The type of disease-causing variants or number of *SMN2* copies entered did not match the patient’s genetic report. In this case, the genetic report information was utilized. If the genetic report with the number of *SMN2* copies was unavailable, the information provided by the patient was not taken into consideration. In total, a report including copy number was available for 263 patients (89.15%).
- Age of onset of the symptoms and maximum motor level reached. This type of discrepancy was more frequent in elderly patients. These results may be due to the time elapsed and the lack of medical reports at the onset of the disease. If a discrepancy between the curator and the patient persisted after communication, this data was marked as invalid and was not included in the database (for statistical extraction).
- Maximum motor level achieved and SMA type. Some patients originally reported their SMA type considering their current motor capacity instead of the maximum motor capacity ever achieved. On occasions, this diagnosis was confirmed by the treating physician themselves. The most common discrepancy detected occurred when the patient stopped walking; sometimes they were erroneously classified as type 2 instead of type 3 SMA.

#### Descriptive analysis

Of the 331 living SMA patients registered by July 2022 in RegistrAME, 295 met the inclusion criteria and were included in the analysis.

These patients ranged in age from 1 to 77 years, with a mean of 20.28 years. Of these, 147 patients were 16 years of age or older and 148 were under 16 years; 136 were female and 159 were male. For analysis purposes, patients ≥ 16 years were considered adults.

When dividing patients by SMA type, 65 (22.03%) were type 1, 143 (48.47%) were type 2, and 85 (28.82%) were type 3. It is noteworthy that there were only two patients registered as SMA type 4, so the inference for this group cannot be very robust. The main demographic and clinical characteristics of the study sample are depicted in Tables 1 and 2.

**Table 1:** Demographic and clinical characteristics according to SMA type

Variable		SMA type 1	SMA type 2	SMA type 3	SMA type 4	Total = 295 N (%)
		*N* = 65	*N* = 143	*N* = 85	*N* = 2	
Age: years **Min-max mean (median)**		1–30 5.74 (4)	2–63 18.67 (17)	3–77 33.82 (34)	57–61 59	1–77 20.28 (16)
		**N (%)**	**N (%)**	**N (%)**	**N (%)**	**N (%)**
Sex	**Female**	28 (43.08%)	64 (44.76%)	44 (51.76%)	0 (0%)	136 (46.10%)
	**Male**	37 (56.92%)	79 (55.24%)	41 (48.24%)	2 (100%)	159 (53.90%)
Functional Status	**Non-sitter**	31 (47.69%)	48 (33.57%)	6 (7.06%)	0 (0%)	85 (28.81%)
	**Sitter**	28 (43.08%)	87 (60.84%)	34 (40%)	0 (0%)	149 (50.51%)
	**Walker**	6 (9.23%)	8 (5.59%)	45 (52.94%)	2 (100%)	61 (20.68%)
*SMN 2* copy number*	**2**	53 (82.81%)	10 (8.4%)	4 (5%)	-	67 (25.48%)
	**3**	11 (17.19%)	103 (86.6%)	52 (65%)	-	166 (63.12%)
	**4**	0 (0%)	6 (5.0%)	24 (30%)	-	30 (11.41%)
Scoliosis Surgery	**No**	56 (86.15%)	66 (46.15%)	69 (81.18%)	2 (100%)	193 (65.42%)
	**Yes**	9 (13.85%)	77 (53.85%)	16 (18.82%)	0 (0%)	102 (34.58%)
Gastrostomy	**No**	39 (60%)	140 (97.9%)	85 (100%)	2 (100%)	266 (90.17%)
	**Yes**	26 (40%)	3 (2.1%)	0 (0%)	0 (0%)	29 (9.83%)
SMN DMT	**Yes**	64 (98.46%)	105 (73.43%)	57 (67.06%)	0 (0%)	226 (76.61%)
	Nusinersen	51	80	54	0	185
	Nusinersen + Onasemnogene abeparvovec	5	0	0	0	5
	Risdiplam	6	25	3	0	34
	Onasemnogene abeparvovec	2	0	0	0	2
	**No**	1 (1.54%)	38 (26.57%)	28 (32.94%)	2 (100%)	69 (23.39%)

*Data available for 263 patients

**Table 2:** Demographic and clinical characteristics according to age

Variable		AGE: Years				
		< 2	2–6	7–10	11–15	> 15
		*N* = 11	*N* = 67	*N* = 37	*N* = 32	*N* = 148
Sex	*Female*	8 (72.73%)	33 (49.25%)	16 (43.24%)	11 (34.38%)	68 (45.95%)
	*Male*	3 (27.27%)	34 (50.75%)	21 (56.76%)	21 (65.62%)	80 (54.05%)
Functional Status	*Non-sitter*	9 (81.82%)	6 (8.96%)	8 (21.62%)	10 (31.25%)	52 (35.14%)
	*Sitter*	2 (18.18%)	43 (64.18%)	23 (62.16%)	18 (56.25%)	63 (42.57%)
	*Walker*	0 (0%)	18 (26.87%)	6 (16.22%)	4 (12.5%)	33 (22.3%)
SMA type	*1*	11 (100%)	36 (53.73%)	10 (27.03%)	3 (9.38%)	5 (3.38%)
	*2*	0 (0%)	25 (37.31%)	22 (59.46%)	24 (75%)	72 (48.65%)
	*3*	0 (0%)	6 (8.96%)	5 (13.51%)	5 (15.62%)	69 (46.62%)
	*4*	0 (0%)	0 (0%)	0 (0%)	0 (0%)	2 (1.35%)
*SMN* 2 copy number*	2	11 (100%)	36 (53.73%)	10 (29.41%)	2 (7.69%)	8 (6.4%)
	3	0 (0%)	31 (46.27%)	23 (67.65%)	19 (73.08%)	93 (74.4%)
	4	0 (0%)	0 (0%)	1 (2.94%)	5 (19.23%)	24 (19.2%)
Scoliosis Surgery	No	11 (100%)	66 (98.51%)	32 (86.49%)	14 (43.75%)	70 (47.3%)
	Yes	0 (0%)	1 (1.49%)	5 (13.51%)	18 (56.25%)	78 (52.7%)
Gastrostomy	No	11 (100%)	51 (76.12%)	30 (81.08%)	32 (100%)	142 (95.95%)
	Yes	0 (0%)	16 (23.88%)	7 (18.92%)	0 (0%)	6 (4.05%)
SMN Treatment	No	0 (0%)	0 (0%)	1 (2.7%)	3 (9.38%)	65 (43.92%)
	Yes	11 (100%)	67 (100%)	36 (97.3%)	29 (90.62%)	83 (56.08%)

*Data available for 263 patients

The initial exploratory analysis revealed that there was practically no missing data for the variables presented, except for the variable “*SMN2* copies”, in which 11% of the values were absent. The *SMN2* copy number value was only considered when the patient provided a valid genetic report. In the event of a patient having two reports with different *SMN2* copy number results, the second report or the one that the treating physician considered appropriate was taken into account. The distribution of *SMN2* copy number is presented in Tables 1 and 2.

Regarding the genetic results, 286 patients have both copies of the *SMN1* gene deleted (in homozygosity), three patients harbor point disease-causing variants in both copies of the *SMN1* gene, and six patients have a deletion of one copy of the *SMN1* gene and a disease-causing variants in the other copy.

### Functional status and motor function

No correlation was found between functional status and SMA type. For example, although the group under 16 years of age had a higher number of patients with SMA type 1 (40.82%), it was the group over 16—where only 3.8% of patients presented SMA type 1—that had the highest number of non-sitter patients (52 adults vs. 33 children).

In patients with SMA type 1, 2, and 3, the three functional statuses (non-sitter, sitter, and walker) were observed (Table 1). In the group under 16 years of age, SMA types 1 and 2 prevailed, while in that of 16 years of age and older, types 2 and 3 prevailed. These types considered less severe were more common in the older age group, however, the motor deterioration over the years revealed a greater impact on patients’ disability. As regards adults, 22.97% were unable to bring their hands to their mouth or had no useful function of their hands. The same disability was present in 6.83% of children under 16 years of age. More than half of the adults could not turn in bed. This contrasted with those under 16 years of age, of whom less than 15% could not turn (Table 3).

Of the 61 walkers (able to walk without support for at least 10 m), 6 patients were type 1, 8 were type 2, 45 were type 3, and 2 were type 4 (Table 1). However, when assessing the quality of gait, no SMA type 1 walker patient was wheelchair-free, either when leaving home or for long stretches. The same was observed in 62.5% (5 patients) and 44.4% (20 patients) of SMA type 2 and type 3 walker patients, respectively.

**Fig. 2:**
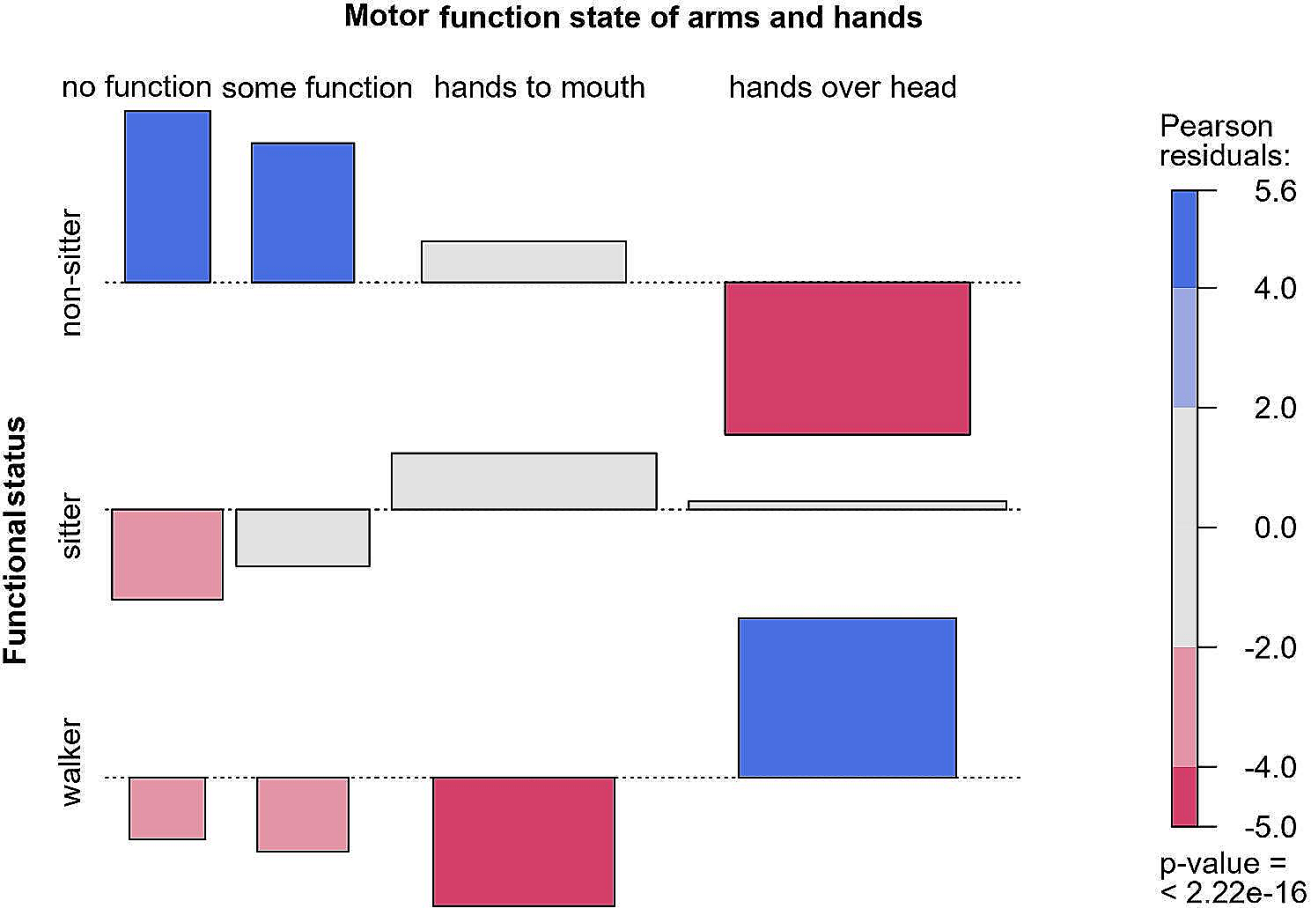
Association between current functional status and motor function in arms and hands

A positive relationship between better Functional Status and better Motor Function States of arms and hands was clearly observed. “Walkers” presented a higher proportion of the motor function “**I can raise my arms above my head**”. “Non-sitters” presented a higher proportion of the motor functions “**No useful function with my hands”** or **“I can’t bring my hands to my mouth, but I have useful function in my hands**”. In this case, the association was statistically significant (*p* < 0.001) Fig. 2.

**Table 3:** Hand Motor Function and Rolling capability according to age

Variable		AGE: Years				
		< 2	2–6	7–10	11–15	> 15
		*N* = 11	*N* = 67	*N* = 37	*N* = 32	*N* = 148
		n (%)	n (%)	n (%)	n (%)	n (%)
Hand Motor Function	No useful function with my hands	0 (0%)	1 (1.49%)	4 (10.81%)	1 (3.12%)	12 (8.11%)
	I can’t bring my hands to my mouth, but I have useful function in my hands	0 (0%)	0 (0%)	1 (2.7%)	3 (9.38%)	22 (14.86%)
	I can bring my hands up to my mouth	5 (45.45%)	5 (7.46%)	7 (18.92%)	15 (46.88%)	71 (47.97%)
	I can raise my arms above my head	6 (54.55%)	61 (91.04%)	25 (67.57%)	13 (40.62%)	43 (29.05%)
Rolling capability	I cannot turn	5 (45.45%)	2 (2.99%)	5 (13.51%)	10 (31.25%)	80 (54.05%)
	I can only partially turn	3 (27.27%)	8 (11.94%)	7 (18.92%)	12 (37.5%)	29 (19.59%)
	I can turn completely	3 (27.27%)	57 (85.07%)	25 (67.57%)	10 (31.25%)	39 (26.35%)

### Scoliosis surgery

From the age of 11 onwards, more than half of the patients had scoliosis surgery, of which 75.49% corresponded to patients with SMA type 2 (Tables 1 and 2).

In total, 13.85% of patients with SMA type 1, 53.85% with type 2, and 18.82% with type 3 underwent scoliosis surgery.

**Fig. 3:**
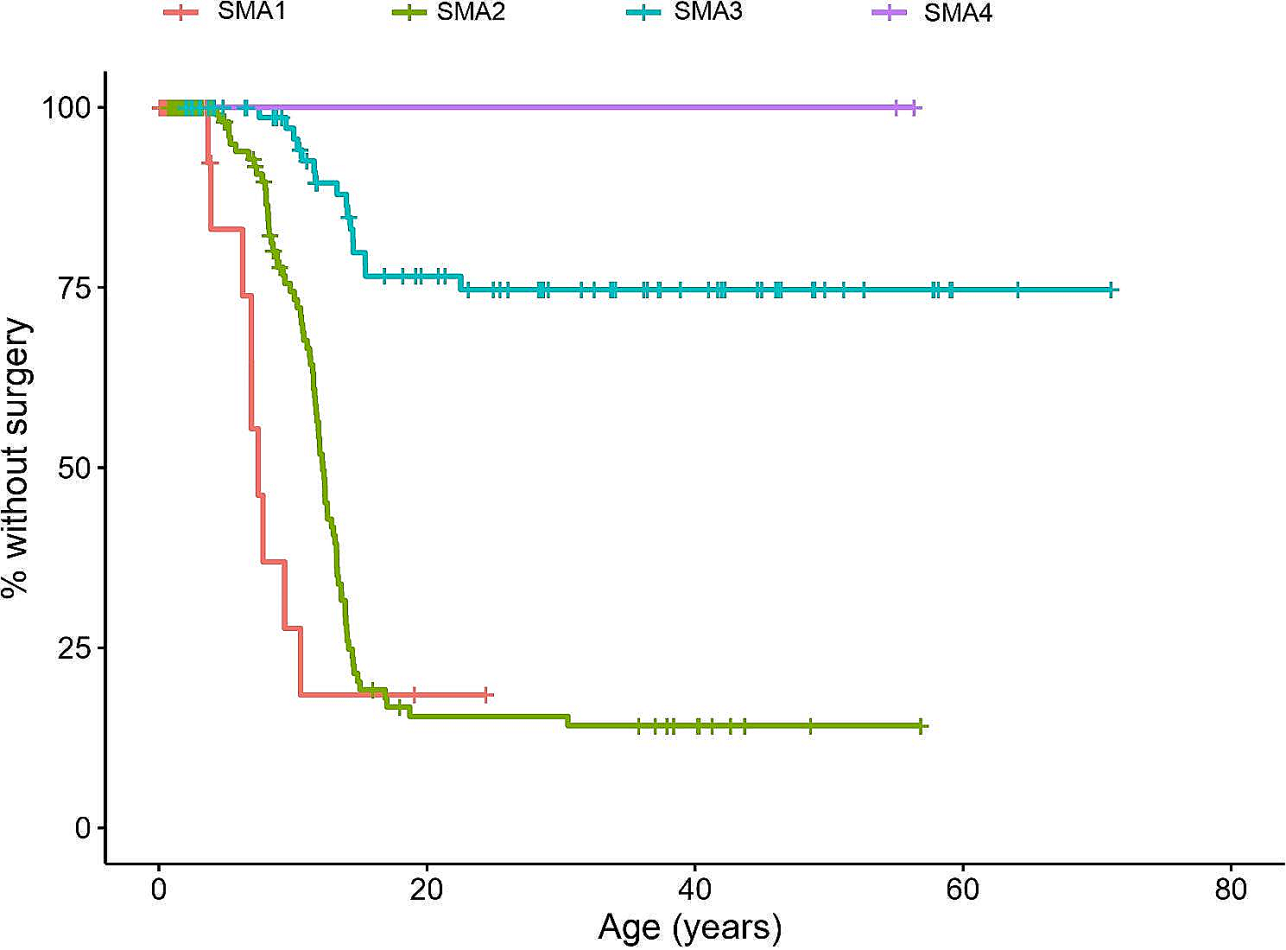
Kaplan-Meier curve until surgery was required depending on SMA type

The relationship between SMA type and time to first surgery was analyzed. The Kaplan-Meier graph shows that patients with SMA type 1 had surgery earlier in life (1st quartile = 6.2 years; median = 7.4 years, 3rd quartile = 10.6 years), followed by SMA type 2 patients (1st quartile = 9.8 years; median = 12.2 years, 3rd quartile = 14.2 years). At a considerable distance were patients with SMA type 3 (1st quartile = 22.5 years) and SMA type 4 (Fig. 3). This association was statistically significant (*p* < 0.001). When only considering the differences between the SMA type 1 and type 2 groups, said differences were not statistically significant (*p* = 0.07).

### Ventilation

Fourteen patients required invasive ventilation. One patient (SMA type 1), who had previously required invasive ventilation stopped using it and, at the time of the study, the patient was on noninvasive ventilation for less than 16 h per day. The use of ventilation is shown in Table 4.

**Table 4:** Need for ventilation

Variable		SMA type 1	SMA type 2	SMA type 3	SMA type 4	Total *N* = 295 N (%)
		*N* = 65	*N* = 143	*N* = 85	*N* = 2	
		N (%)	N (%)	N (%)	N (%)	
**Invasive Ventilation**	Yes, part of the day*	8 (12.31%)	1 (0.7%)	0 (0%)	0 (0%)	9 (3.05%)
	**Yes, all day**	5 (7.69%)	0 (0%)	0 (0%)	0 (0%)	5 (1.69%)
**Noninvasive Ventilation**	Yes, part of the day*	37 (56.92%)	78 (54.55%)	7 (8.24%)	0 (0%)	122 (41.36%)
	**Yes, all day**	1 (1.54%)	0 (0%)	0 (0%)	0 (0%)	1 (0.34%)
**Never use any type of ventilation**		14 (21.54%)	64 (45.45%)	78 (91.76%)	2 (100%)	158 (53.56%)
Mean (SD) / n (%)					Median (1st, 3rd Q.)	
Age of onset of invasive ventilation		1.92 (3.41)	15 (-)	-		2.85 (4.79)
		0.83 (0.5, 1.17)	15 (15, 15)	-		0.88 (0.52, 1.79)
Age of onset of noninvasive ventilation		1.22 (1.6)	11.58 (11.83)	33.86 (18.61)		9.63 (12.83)
		0.5 (0.33, 1.19)	8 (3, 14)	35 (26.5, 43.5)		4.5 (1.31, 12)

*Less than 16 h per day

Since the number of patients requiring invasive ventilation was not very high (14 of the total; 4.7%), no additional subgroup analysis was performed.

Thirteen patients with SMA type 1 required invasive ventilation and 38 required noninvasive ventilation. The registry does not include the reason why noninvasive ventilation was prescribed (proactive treatment or treatment).

As for children, 21.43% (i.e., 14 children) with SMA type 1 aged between 1 and 7 years (mean 2.75 years) did not use any type of ventilation; eight were sitters, five were non-sitters, and one was a walker (this patient walked at home, when leaving home they used a wheelchair). Twelve of these children had two *SMN2* copies.

**Fig. 4:**
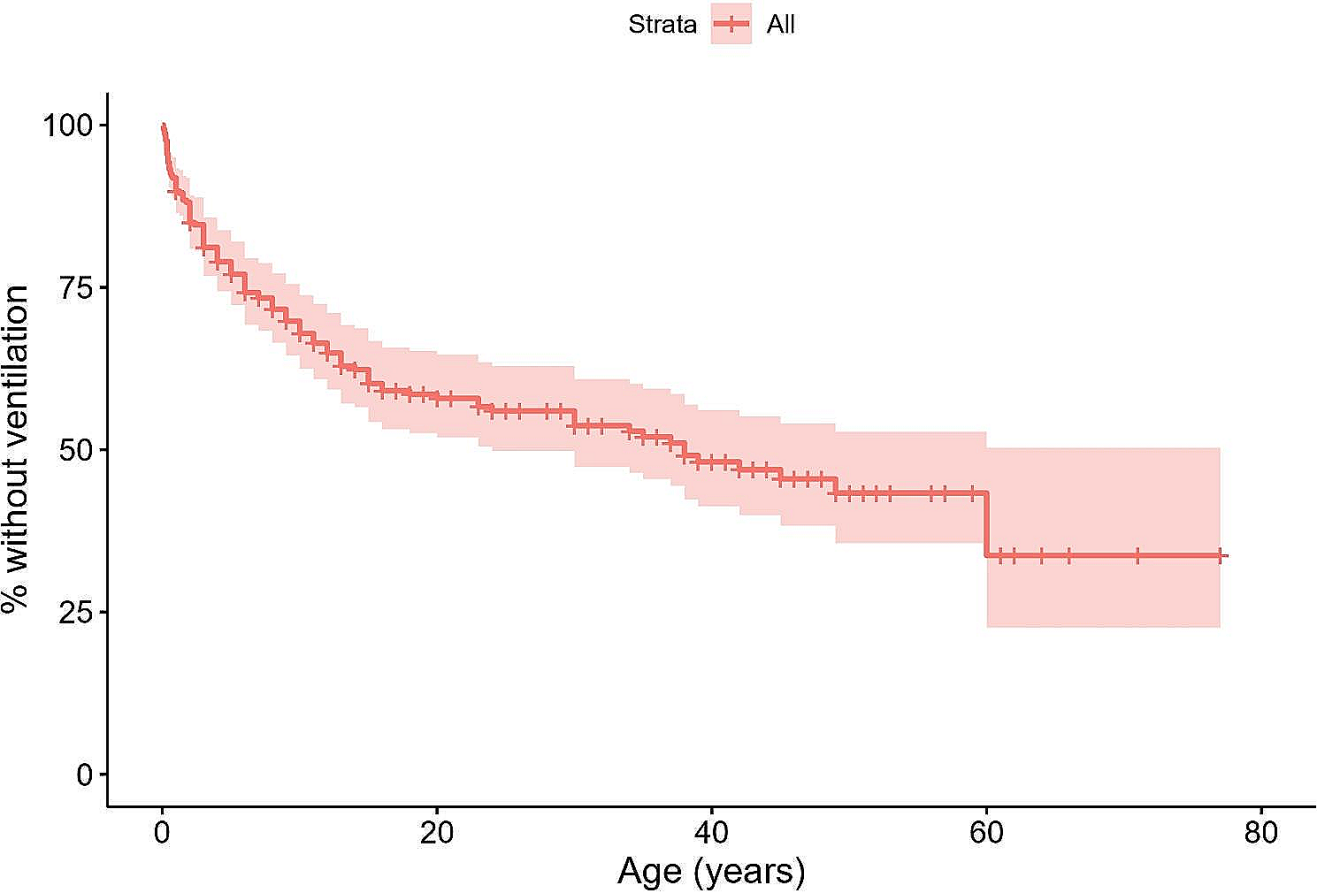
Kaplan-Meier curve over time (age in years) until the initiation of noninvasive ventilation

**Fig. 5:**
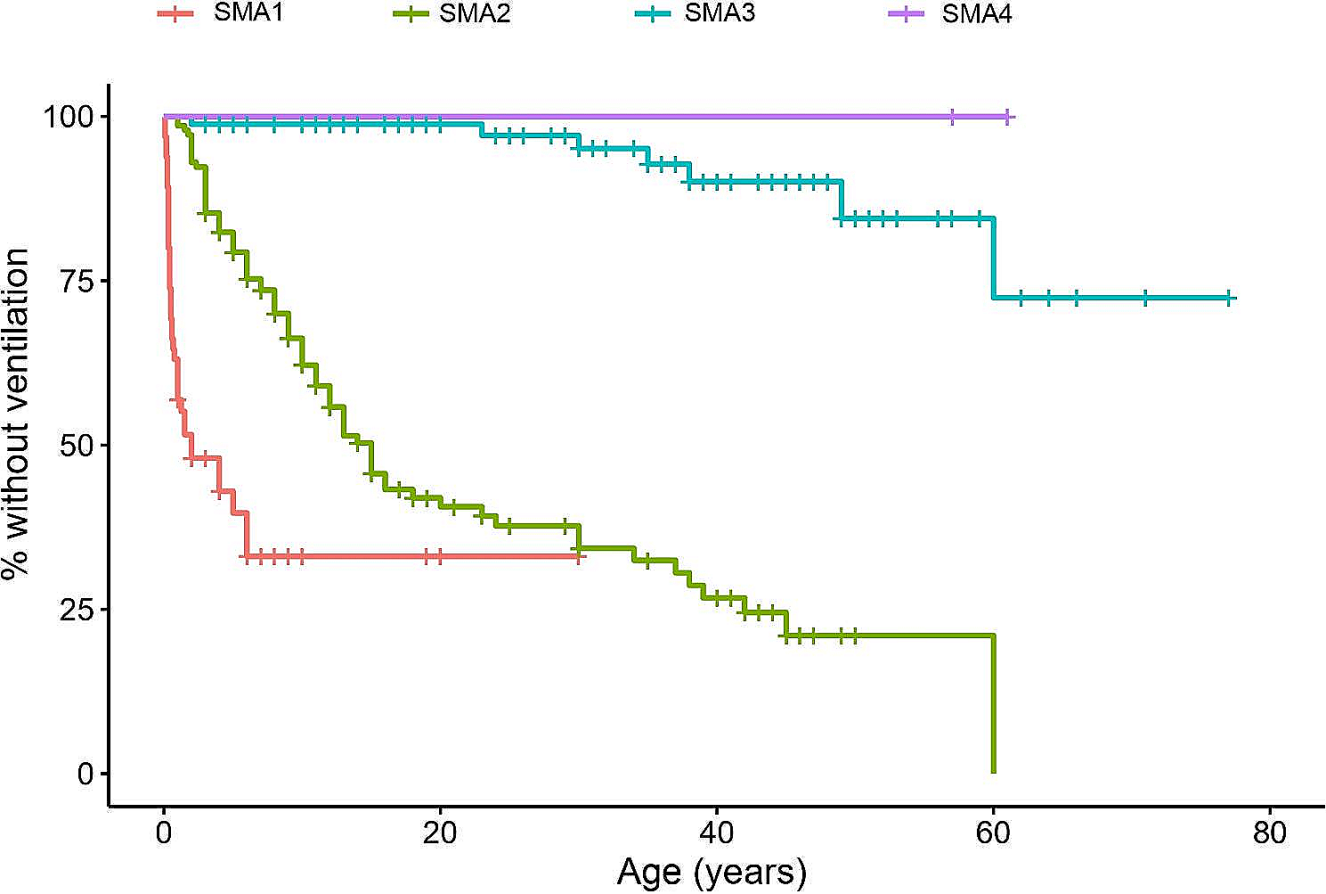
Kaplan-Meier curves over time (age in years) until the initiation of noninvasive ventilation according to the type of SMA

**Fig. 6:**
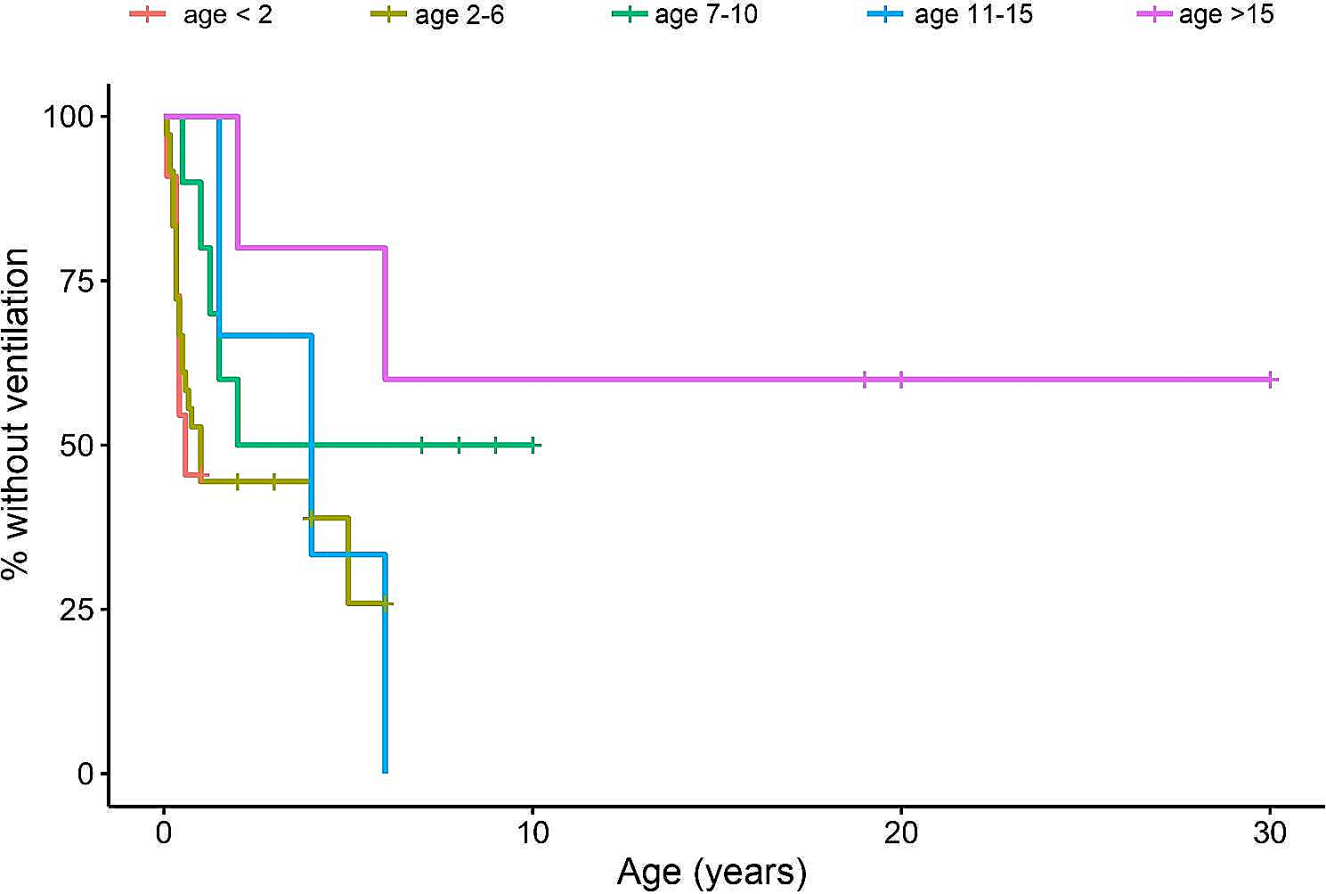
Kaplan-Meier curve over time (age in years) until the initiation of noninvasive ventilation according to age group

A Kaplan-Meier curve estimate analysis was performed to study the time elapsed until noninvasive ventilation was prescribed. In addition, Fig. 4 shows the overall Kaplan-Meier curve, Fig. 5 shows the curves according to each patient’s SMA type and Fig. 6 shows the curves according to age. It is clearly observed that patients with SMA type 1 were the first to initiate ventilation treatment (1st quartile = 0.4 years; median = 2 years), followed by SMA type 2 (1st quartile = 7 years; median = 15 years) and, at a great distance, SMA type 3 (1st quartile = 60 years) and SMA type 4. The differences were statistically significant (*p* < 0.001).

### Gastrostomy

Twenty-nine patients (9.83%) had a gastrostomy, of which twenty-six (89.66%) were SMA type 1. Of the 29 gastrostomy patients, 13 had invasive ventilation, and one patient no longer required invasive ventilation.

40% of patients with SMA type 1 had a gastrostomy (2–30 years; mean: 8.12, median 6 years). Reports of *SMN2* copy number were available in 25 of the 26 types 1 patients with gastrostomy. Twenty-one patients (84%) had two *SMN2* copies but only four gastrostomy patients had three *SMN2* copies (10–21 years; mean: 15 years). The relationship between *SMN2* copy number and gastrostomy requirement was not statistically significant (*p* = 0.84). Of the patients with three *SMN2* copies, the age at gastrostomy was between two and eight years, and none started DMT before the age of five years (mean: 11 years).

## Discussion

The Spanish Spinal Muscular Atrophy Registry -RegistrAME- has been active in Spain since 2015, encompassing a substantial population of both adults and children with SMA. This represents the first report utilizing data from RegistrAME, providing real-world insights into the largest cohort of individuals with SMA in Spain published to date.

RegistrAME relies on the proactive participation of the patients, supervised by a specialist. It combines direct patient experiences with clinical data curated by medical specialists. It promotes patient empowerment and participation while providing the highest quality clinical data.

The value of a registry depends mainly on the quality of the data contained within it, which will be determined by the type of data collected and how it is verified [19]. The data must be of excellent quality, therefore, it must be complete, consistent, unambiguous or discrepant, validated, and with high-quality curation. In the case of a self-reported registry, data is reliable as long as it is validated via a rigorous curating system.

Data in self-reported registries are not supposed to be authenticated by clinicians, however, in our experience, as already mentioned for other registries, at least the accuracy of the genetic information and clinical diagnosis must be guaranteed [21]. Patients are not trained clinicians who always know the correct answer to clinical and genetic questions with closed answers and, therefore, they require assistance.

In the case of the Spinal Muscular Atrophy Registry, in addition to genetic validation (type of disease-causing variants/deletion, *SMN2* copy number), it is important to confirm the type of SMA depending on the maximum motor level reached—without DMTs—and the age of onset of symptoms. Despite having a userfriendly platform, it is necessary to provide the patient with assistance via an adequate communication system since patients are a very heterogeneous population in terms of age, understanding of the disease, and their competence in the use of new technologies. This type of assistance is even more important for the adult patient, who grew up in an environment where current treatment options were not available.

Self-reported registries offer several advantages. Firstly, their universality is a key benefit as they are not confined to specific institutions or patients under observation at a specific center. The regularity of updates is facilitated through direct and ongoing contact with patients, allowing for the collection of Patient-Reported Outcomes (PROs) and data on functionality, autonomy, and quality of life. These supplementary insights complement clinical data and hold increasing significance. Moreover, in our case, with the patient organization serving as the registry holder, the dataset is inherently patient-centered, guaranteeing meaningfulness and relevance for individuals affected by the condition. Furthermore, it employs terminology accessible to patients. The drawback of this type of registry is that it does not capture other types of clinical data, such as motor scales or clinical observations. There may also be a selection bias based on the type of patients who choose to participate, as they may have different characteristics compared with those who do not participate.

In this study, we present a snapshot of the situation of a large cohort of SMA patients, both adults and children. Of the 295 patients studied, 147 were under 16 years of age and 148 were 16 or older. In contrast to that observed in other registries [22, 23], in RegistrAME, type 2 and not type 3 was the predominant SMA type (48.87% and 28.81%, respectively).

Unlike a previous Spanish study [5], there were no patients with just one *SMN2* copy in our registry. This is probably because there were no patients with SMA type 0, type 1 A, or with one copy of *SMN2* in RegistrAME. For ethical reasons, they are not invited to participate in RegistrAME due to the poor prognosis of these children and the lack of access to treatment in Spain. Another difference observed between the two studies was the presence in RegistrAME of SMA type 2 patients with four *SMN2* copies (5%).

All children under two years of age harbored two *SMN*2 copies compared with only 6.4% of those over 16 years. This is a reflection of the impact on survival of *SMN2* copy number as a phenotype modifier. Copy number is an important phenotype modifier, however, it is not sufficient to predict the phenotype [24] and should not be used as a gold standard for predicting disease severity. Another consideration is the fact that *SMN2* copy number reports can be, in many cases, erroneous [25]. This fact was also observed in RegistrAME, in which several patients had different *SMN2* copy number reports. Even though *SMN2* copies are usually performed in expert centers, it is possible that some determinations were not accurate due to technical limitations [25, 26]).

Our results confirm previous studies reporting great phenotypic variability in the different SMA types [27]. In addition, the current classification of SMA types to represent the severity of the disease does not consider the progression of the disease over the years. As a consequence, we found affected patients with a theoretically less severe condition who were suffering a greater impact on loss of motor functions and physical disability. Almost a quarter of adults were not able to reach their mouth with their hands or had no useful function of their hands, and more than half could not turn in bed. This contrasts with those under 16 years of age, of whom the same disability of the upper limbs was observed in less than 7%, and 14.97% were unable to turn in bed.

All functional statuses can be observed for each SMA subtype 1 to 3. There was also great heterogeneity within each functional status. These differences must be taken into account when managing family exceptions. For example, 49.18% of patients considered walkers, never used a wheelchair; however, 50.82% of them had to use it when they left their home or needed to cover a long distance. In our registry, no patients with SMA type 1, as well as most type 2 patients who achieved gait, were free from the use of a wheelchair when leaving home or for long walks.

This great phenotypic variability in the subtypes reinforces the need to achieve a consensus for a classification that reflects the real state of the individual [27]; to do so, functional status, age of treatment initiation, and *SMN2* copy number must be included within the classification [28]. This classification would allow for a more realistic approach to the impact on disability.

Patients with SMA type 2 were more likely to undergo scoliosis surgery compared with SMA type 1 patients (53.85% vs. 13.85%). However, this scenario might be a consequence of the patients’ age in the SMA type 1 group, as the majority (87.65%) were under 11 years old, and 72.31% were younger than seven years old, so this situation may change drastically as this population ages. Although not statistically significant, children with SMA type 1, on average, had their first scoliosis surgery about five years earlier than patients with SMA type 2. If the need for surgery at such an early age is confirmed in a larger sample, it would highlight the need to know the evolution over time and the impact of surgery on these children at such a young age. It is necessary to establish standard-of-care guidelines for scoliosis in patients with SMA type 1 in the treatment era.

It was surprising that 21% of patients with SMA type 1 (1–7 years old; mean: 2.75) did not use any type of ventilation (because of their age, most of these patients were diagnosed when the first treatment was already available). In addition to the significant impact of the treatments, this may point to a new respiratory approach, in which the management of respiratory care is less proactive.

40% of patients with SMA type 1 had a gastrostomy tube and, of these, 84% had two copies of *SMN2*. The correlation between copy number and the need for gastrostomy was not statistically significant, however, patients with three copies (10–21 years) might have been influenced by other factors, such as late initiation of treatment, as well as greater disease progression at the time the gastrostomy procedure took place.

More than half of type 1 SMA patients with a gastrostomy were six years old or younger, because of their age, these patients were diagnosed when treatments were already available. This indicates the continued high impact of bulbar involvement in the posttreatment era [29] and the implementation of standards of care in the management of swallowing. Bulbar and respiratory function need to be closely monitored [30]. It is important to take this “realworld” information into account when managing the expectations of families with an SMA type 1 member. Nowadays, early diagnosis of swallowing problems is playing a very relevant role in the followup of this population, and new tests permitting a good evaluation have been developed [31], even when goldstandard tools, such as videofluoroscopy, are not available.

### Limitations of the study

Data are collected from a selfreported patient registry and not all patients have the same proactivity when it comes to participating in a registry, which may affect the profile of the patients evaluated.

## Conclusion

Increased knowledge of the evolution of SMA patients in the “real world” allows for a more accurate identification of the impact of the disease. The experience with RegistrAME also includes patient-relevant data and a better understanding of unmet needs. With this information, it is possible to develop better standards of care and a more adequate approach to patient and family expectations.

## Data Availability

All data generated or analysed during this study are included in this published article.

## Abbreviations

DMT: Disease-modifying therapies
PROS: Patient-Relevant Outcomes
SMA: Spinal Muscular Atrophy
SMN: Survival motor neuron
